# Coaching and/or education intervention for parents with overweight/obesity and their children: study protocol of a single-centre randomized controlled trial

**DOI:** 10.1186/s12889-019-6640-5

**Published:** 2019-03-28

**Authors:** Shazya Karmali, Victor Ng, Danielle Battram, Shauna Burke, Don Morrow, Erin S. Pearson, Patricia Tucker, Tara Mantler, Anita Cramp, Robert Petrella, Jennifer D. Irwin

**Affiliations:** 1 Western University, 1151 Richmond St. (Arthur & Sonia Labatt Health Sciences Building), London, ON Canada; 2 Health & Rehabilitation Sciences, 1151 Richmond St. (Arthur & Sonia Labatt Health Sciences Building), London, ON Canada; 3 Department of Emergency Medicine, Scarborough Health Network - Centenary Campus, 2867 Ellesmere Road, Scarborough, ON Canada; 4 Food and Nutritional Sciences, Brescia University College, 1285 Western Road, London, ON Canada; 5 School of Health Studies, 1151 Richmond St. (Arthur & Sonia Labatt Health Sciences Building), London, ON Canada; 6 School of Kinesiology, London, ON Canada; 7 School of Kinesiology, Faculty of Health and Behavioural Sciences Sanders Fieldhouse, Lakehead University, Thunder Bay, Canada; 8 School of Occupational Therapy, Elborn College, 1201 Western Road (Elborn College), London, ON Canada; 9 Middlesex London Health Unit, Healthy Living Department, 50 King St, London, ON Canada; 10 Schulich School of Medicine and Dentistry, 1151 Richmond St, London, ON Canada

**Keywords:** Behavioural intervention, Overweight/obesity prevention, Overweight/obesity treatment, Parent-child, Coaching

## Abstract

**Background:**

In Canada, a majority of children and adults are insufficiently active for health gains, and about one in seven children and over 20% of adults are overweight or obese. Overweight and obesity are risk factors for many chronic diseases in both adults and children and can result in lower quality and quantity of life. Children whose parents are overweight or obese are more likely to become overweight themselves. Thus, parent/child interventions are important for reducing obesity and promoting long-term healthy weights among members of the family unit. Programs using Co-Active coaching have resulted in positive behaviour changes among adults with overweight/obesity; however, little research has explored the effects of Co-Active coaching on parents, and the consequent impact on the family unit (i.e. all parents and children in the same household). This protocol paper provides a detailed methodological account of a coaching-based program targeting parent and child dyads, in hopes of enhancing health behaviours within the family unit.

**Methods:**

Using a randomized controlled trial design, the researchers aim to identify the impact of coaching plus education (intervention) compared to education only (control) on parents with overweight/obesity and their children (ages 2.5–10, of any weight). A total of 50 dyads are being recruited and randomly assigned using a 1:1 ratio into the control or intervention group. The control group receive 6 webinar-based education sessions focused on physical activity and nutrition. The intervention group receive the same education sessions and nine, 20-min telephone-based sessions with a certified coach. Coaching and health education sessions are conducted with the parent/guardian of the dyad. This paper provides a detailed methodological account of this program.

**Discussion:**

The expected findings from this research will advance coaching literature, research, and practice on this topic by determining whether coaching and education are more effective than education alone at producing behaviour changes among a family unit. If proven effective, this approach may be applied more broadly through public health interventionists to parent and child populations in hopes of affecting change with both individuals and their families.

**Trial registration:**

ISRCTN ISRCTN69091372. Retrospectively registered 24 September 2018.

## Background

Currently, fewer than 10% of Canadian children meet the guideline of 60 min of moderate-to-vigorous physical activity (MVPA) per day, while only 15% of Canadian adults meet the guideline of 150 min of MVPA per week [1]. In addition, it has been reported that Canadian adults with children are less active than those without children [1]. Long-term persistence of these behaviours can result in the development of overweight/obesity in both adults and children, in turn causing the development of adverse health conditions, which are generally preventable [2, 3]. Overweight/obesity in early life stages continues into adolescence and adulthood, and can result in lifelong struggles with physical, mental, and social health (e.g., asthma, blood pressure, weight-based stigmatization, behavioural problems, and low self-esteem [3–5]).

Family and home environments shape early health habits, and parents play a crucial role in developing their children’s nutrition and physical activity (PA) behaviours, in that parents determine the types of foods available in their homes and provide opportunities to be active (or inactive [6]). Therefore, it is important to encourage and support the development of healthy behaviours within the family unit, and to provide parents with resources and knowledge to promote these healthy habits.

Interventions designed to prevent and/or reduce obesity in adults and children, should engage both parent and child in order to develop healthy behaviours within the family unit overall. Thus, the researchers of this project sought to determine the impact of coaching and/or education on parents with overweight/obesity and the consequent impact on their children (aged 2.5–10, of any weight).

### Pediatric and adult overweight/obesity and associated adverse effects

#### Pediatric overweight/obesity

American data reveals that one quarter of preschool-aged children have overweight or obesity; in Canada, 8.5% of children aged 5–9 years and 12.9% of 10–14 year-olds experience obesity [7, 8]. In the same age groups, the rates of overweight are 15.4% (5–9 year-olds), and 23.0% (10–14 year-olds [8]). Males aged 5–11 were reported as significantly more likely to have obesity than females in the same age group [9, 10]. These are alarming statistics for a problem that is preventable if supportive and sustainable lifestyle tools are in place.

The increase in obesity rates also has caused a drastic increase in the incidence of adult obesity-related diseases, such as Type 2 diabetes, cardiovascular disease (CVD), obstructive sleep apnea, and mental disorders in children [11]. Children with overweight/obesity are more likely to develop CVD compared to adults, and subsequent weight loss may not eliminate that excess risk [5]. Conditions such as elevated blood pressure, insulin resistance, and dyslipidemia (i.e., an abnormal amount of lipids in the blood) are being diagnosed in children, prompting the growth of new specialties and clinics targeted toward treating hypertension, Type 2 diabetes, and fatty liver in pediatric populations [5]. Children with obesity are 3 times more likely to develop hypertension than their normal-weight counterparts, and 40% of children with overweight will continue to have increased weight during adolescence [3]. In fact, up to 80% of adolescents with obesity will maintain this weight status into adulthood [3].

Obesity is related significantly to feelings of shame in children, which may affect the development of the child’s personality, identity, and socialization, and can result in decreased pursuits of higher education [12]. A review conducted by Hamilton and colleagues [13] outlined that, in the United States, 25 to 31-year-old adults who experienced obesity as adolescents earned 7.5% less than their counterparts who did not have obesity as adolescents. Lifetime healthcare costs and income penalties were greater in females who experienced obesity as adolescents, while costs due to workdays lost were greater in males who experienced obesity as adolescents [13]. In addition, there seemed to be proportionality between BMI and costs, in that lifetime costs (i.e. healthcare costs and productivity loss costs) increased in proportion with excess weight in childhood or adolescence [13].

The above overview underscores the intensity of overweight/obesity-related issues for children, and the importance of encouraging and developing early healthy bodyweight behaviours among children of any weight [14, 15]. As outlined below, parents’ own obesity status and behavioural prompts are key influencers in the development of their children’s healthy behaviours.

#### Adult overweight/obesity

In 2014, 20.2% of Canadians 18 and older (roughly 5.3 million adults) reported height and weight that classified them as obese, and 40.0% of men and 27.5% of women were classified as overweight [10]. Similar to childhood overweight/obesity, the condition in adulthood is a risk factor for developing poorer health outcomes such as diabetes, heart disease, stroke, and some types of cancer [16]. The psychological effects of obesity are among the most underestimated consequences of this disease; these include reduced quality of life, bullying, negative self-esteem, increased anxiety, risk of isolation, and worsening depression [12].

The causes of obesity are complex, and include an interaction of biology/genetics, behavioural, social, and environmental factors that result in excess weight [17]. In order to encourage sustainable healthy behaviour changes, it is important to implement lifestyle interventions that address a variety of risk factors [17].

### Importance of involving parents in childhood obesity prevention/interventions

Dietary and PA habits established during the early years determine the progression of obesity later in life [18], Given children’s parents, home, and family environments are among the strongest influences on their health behaviours, an optimal method for promoting and encouraging positive behaviour change is targeting, as a dyad, parents *and* their children during their formative younger years [18–21].

The odds of becoming obese as an adult are doubled for children under age 10 who have at least one obese parent, and the probability of childhood obesity persisting into adulthood is estimated to increase from approximately 20% at age 4 years to approximately 80% by adolescence [22]. Therefore, interventions that require only the adult dyad member to be overweight or obese provide a suitable approach.

Children’s obesity-related behaviours are influenced by parental knowledge (how to cook healthy meals), attitudes (valuing PA), modeling (being active themselves), support (financial, logistical, participating with their child), and encouragement [1, 23–26]. In fact, parental role modeling and support for PA are independently associated with their children’s PA levels [1]. Furthermore, when compared to interventions targeting children only, programs that also engage parents are associated with higher self-esteem among the children participants [27].

Previous research examining the influence of family on childhood overweight/ obesity has focused predominantly on parent-centered, unidirectional aspects of parenting (i.e., what the parent does or believes), including maternal feeding practices, and parental PA and nutrition knowledge [6]. Specifically, childhood obesity researchers have measured primarily characteristics and/or behaviours of the parent and child individually [18]. More recently, researchers have examined parents’ actions as well as how they complete these actions, by investigating relationships between parenting styles and young children’s weight status [18]. The direction of developmental research has also shifted to viewing the parent and child as a unit or dyad as opposed to as individuals, thereby examining and encouraging an interactive perspective on the parent-child relationship [18, 28]. This interactive standpoint reflects the relations between parents and their children and demonstrates that children’s development is shaped by the reciprocal nature of both parent- and child-level factors [18, 28].

A further reason to communicate with parents about their children’s health behaviours is to increase parental awareness regarding the risks associated with children developing overweight/obesity and its adverse conditions, and to encourage parents to take action toward promoting healthy behaviours [29]. Parents who recognize excess weight in their children as a health risk may be more motivated to encourage healthy behaviour change than parents who do not [30, 31]. Thus, education and support for parents is a fundamental step to promoting healthy behaviours in children and their families [21].

Although child involvement is important in family-based initiatives, the parent controls implementation of the treatment/intervention [32]. Thus, successful completion of program tasks is mostly determined by the parents’ motivation for participation [32]. Obesity prevention researchers have shown that parental motivation is significantly associated with encouragement of healthy behaviours (i.e. dietary and PA changes) in their children [32–34]. Therefore, it can be hypothesized that parental characteristics, particularly motivation to participate in and complete a behaviour change program with their child, are important in the development of obesity prevention initiatives for the family unit [32]. Thus, the researchers of the current paper have included measures to assess parental motivation to engage in healthy behaviours.

With regard to the most effective methods of targeting parents, Wolfenden and colleagues [21] reported that a random sample of parents of children between the ages of 2–15 expressed a preference for, and increased use of, low-intensity interventions, such as the delivery of information through mail or email. In addition, telephone and internet-based services were viewed as intensive and interactive support that parents would be most likely to use [21].

The research summarized above identifies a need for behaviour-based strategies to focus on modifying family lifestyle patterns to prevent and reduce the prevalence and impact of obesity and its associated health consequences on parents and their children. In order to promote behaviour change within the family unit, the method of education and/or Co-Active coaching was selected because of their success in developing sustainable behaviour change within individuals. The importance of considering empowerment when using parent-child dyads as a strategy to facilitate and sustain behaviour change has yet to be fully explored, and Co-Active coaching may prove to be an effective approach.

### Co-active coaching

Some coaching definitions and training programs posit that the primary purpose of coaching is to advise clients and is based on a relationship where the coach is viewed as the ‘expert’ [35]. In terms of health-related behaviour change, some studies consider a coach to be any support person who coaches an individual who is living with an illness of health issue [35]. However, coaching does not represent a specific phenomenon, but instead connotes a behavioural intervention with many dimensions and styles [35]. As such, it is important to clearly identify the method of coaching employed when attributing behaviour change to coaching [35].

Co-Active coaching [36] involves an alliance between coach and client, using key behavioural elements including self-efficacy, acknowledgement, goal-setting, personal values, and empowerment [36, 37]. The premise of Co-Active coaching does not center on solving problems, though problems may be solved through the process, but is a way of effectively empowering people to find their own answers [36]. The client is viewed as the expert on his/her own life and has the answers – albeit, often not concretized prior to coaching – to their own life questions, thus empowering the client to create their own solutions to their identified problems [36]. The term ‘Co-Active’ refers to the fundamental nature of a coaching relationship, in which the coach and client are active collaborators, and create an alliance in order to meet the client’s needs [38]. This partnership between coach and client seeks to meet the needs and learning style of the client, which, in turn, strengthens the client’s ability to self-manage his/her behaviours and attitudes based on his/her own values [36].

Co-Active coaching is an accredited coach-training method recognized by the International Coaching Federation [38]. The Co-Active coaching accreditation program spans approximately 12 months and is comprised of 5 in-person experiential workshops, followed by a six-month certification program [38]. The Coaches Training Institute [38] and Kimsey-House and colleagues [36] outline the three foundational principles of Co-Active coaching, which are: fulfillment (indicating life satisfaction); balance (based on using different perspectives to view situations and make meaningful choices); and process (fully experiencing any given moment). This Co-Active model is based on the client’s agenda, and the relationship between coach and client is tailored to the communication approach that works best for them [37–39].

### Co-active coaching and adult obesity

Researchers have established that interventions targeting health behaviour change should be based upon tested theories, and that these theories encompass the psychological and structural processes that are assumed to guide and regulate behaviour [40–42]. The Co-Active coaching approach has been grounded in several well-established behavioural theory frameworks [35, 39], such as Social Cognitive Theory [43], the Theory of Reasoned Action [41], and the Theory of Planned Behaviour [44]. Co-Active coaching researchers have demonstrated this approach’s effectiveness in producing positive behaviour change in a variety of health-related areas, such as PA, nutrition, and smoking cessation, and more germane to this study, overweight/obesity [45–48].

Co-Active coaching has been evaluated as an effective short- and long-term obesity reduction approach in adult populations, and shown positive results, including reductions in BMI and improvements in relevant psychosocial variables such as self-esteem and functional health status [47–49]. While an increasing number of studies have underscored the need to explore parent-focused or family-based childhood obesity prevention interventions [23, 28, 45, 50, 51], no studies have explored the utility of a coaching approach, such as Co-Active coaching, within a parent- or family-focused obesity reduction/healthy bodyweight promotion intervention aiming to impact both a parent and child concurrently. The value of coaching as a healthy weight intervention for adults, the necessity of incorporating parents in children’s health interventions (e.g., [52–55]), and supporting parent-child dyads is vital for sustained health behaviour change has been clearly outlined in previous research [18, 20, 22].

### Study purpose

This study will explore the impact of a parent coaching intervention, with parent and child outcomes, on promoting healthy behaviours among the dyad. Specifically, this research aims to identify the impact of a coaching plus health education intervention compared to health education only on: (a) the PA levels of children (ages 2.5–10) and their parents with overweight/obesity; (b) the dietary intake of children and their parents with overweight/obesity; (c) parental motivation to engage in healthy behaviours; and (d) parental perspectives on how the program has impact on their and their child’s nutrition and PA behaviours. These primary outcomes will be measured via in-person parent interviews, parent and child 7-day step count and 24 h dietary intake, and standardized and validated questionnaires. Due to children’s active growth periods, PA and nutrition behaviours can be better predictors of health than anthropometric indicators [54]. Secondary outcomes will be measured by assessing: (a) parental BMI (calculated by measuring weight in kg, over height in m^2^); (b) parents’ overall perception of health; and (c) parents’ psychosocial variables (i.e., social support, self-esteem, and self-efficacy).

The research team hypothesizes that immediately following and at 6 months post-intervention, the coaching plus education group will report higher levels of parent-child PA, greater improvements in parent-child dietary intake choices, greater parental psychosocial benefits, and greater improvements in parents’ BMI values, compared to those who receive education only. The research team predicts that parents from higher socioeconomic status (SES) backgrounds will be impacted more favorably than those from lower SES backgrounds, as researchers have found greater difficulties associated with affecting behaviour change within lower SES environments [20]. It is also hypothesized that male parents and children will have higher PA levels, while female parents and children will have greater dietary intake improvements, and female parents’ psychosocial health will be impacted most positively, given that previous research has found young males tend to be more active than their female counterparts, and obesity affects psychosocial health of women more negatively than men [27].

The purpose of the current protocol paper is to provide a detailed methodological account of this parent-child study with a view toward informing future coaching and obesity prevention/treatment programs designed to impact health behaviours positively within the family unit.

## Methods

### Study design

This 3-month, single centre, randomized controlled trial (in accordance with SPIRIT guidelines [56]) is currently underway and aims to improve PA and nutrition behaviours within the family unit. Using a single blind, block randomized design via computer randomization (using an online random sequence generator), parent-child dyads are assigned to either: coaching plus health education (intervention) or health education alone (control). The blocked randomization design ensures an equal proportion of dyads are assigned to each group. Only the lead researcher is aware of allocation assignment; parent participants are made aware of their group assignments at their baseline appointments.

#### Sample size and eligibility criteria

A sample size calculation was conducted using the Horatio Computer Software program [57]. The inclusion of 50 parent-child dyads was deemed sufficient to detect a large effect size (d = 0.8) of a two-level, between groups independent variable, 79% of the time, using a 0.05 alpha level.

To be eligible for this study, parents/guardians must have a BMI of > 25 kg/m^2^, live with their child (aged 2.5–10) for at least 5 days of the week, speak English, and are comfortable using a computer for data collection purposes. Because adolescence spans 10–19 years [58], the research team decided targeting children aged 10 and under for the study described in this paper would be the most impactful. If there are two parents and two children in a family who meet the inclusion criteria, they are permitted to participate as two separate dyads. In the case where two parent-child pairs within the same household are both randomized to receive the intervention, the parents will work with two different coaches.

#### Certified professional co-active coaches (CPCCs)

A total of 16 certified CPCCs have been recruited through the research team’s network to deliver the intervention (three coaching sessions per month, for three months) to parents assigned to the intervention group. The lead researcher informs interested coaches about the study design and outcomes being measured and answers any questions or concerns coaches may have. Coaches involved in the study are assigned between 1 and 3 participants based on how many they feel they can work with over the duration of the study. Upon completion of the intervention, coaches receive an honorarium for each participant with whom they have worked.

It is important that all coaches are certified in Co-Active coaching because: (a) it is an accredited method of coaching, (b) it is consistent with respect to training, in that all coaches are taught the same way and use consistent tools; and (c) this particular coaching method has been shown to be effective in changing health behaviours [35].

### Participants and recruitment

Ethical approval has been obtained from the Office of Research Ethics at the host institution. Participants are being recruited via poster advertisements at various locations including: clinics and medical offices, childcare centers, pharmacies, the local health unit, Young Men’s Christian Association (YMCA) sites, libraries, recreation centers, Ontario Early Years Centers, and community organizations (including local businesses). In addition, a radio advertisement is airing, and recruitment posters are advertised on Facebook groups/pages, Twitter, Kijiji, and in a local parenting magazine. Once participants contact the researcher, the study is explained in more detail, and the researcher asks several questions to determine eligibility. When a parent-child dyad is determined as eligible to participate, a baseline appointment is made in order to conduct the parent’s measurements (height, weight, and waist circumference), inform the parent of group assignment, sign consent forms, provide the dyad with pedometers, and further explain how the study will unfold.

#### Data collection

Rolling enrollment has been adopted, making the data collection periods tailored to each individual in the study. Data are being collected at baseline (i.e., one week prior to the start of the intervention); six weeks into the intervention; immediately post-intervention (i.e., three months); and six months post-intervention. All data is being entered electronically at the host university, on a secure server. Consent and other written forms are stored and locked in a secure drawer, in a locked office, at the host university. In order to maintain confidentiality and anonymity, participants are assigned a unique identification code upon registering in the study. Participant files will be kept in storage for up to 5 years after completion of the study. Baseline and follow-up assessments are conducted at the host university or at the participant’s home by the lead researcher and a research assistant. An email link is sent to parent participants asking them to complete the questionnaires, and email and telephone reminders are conducted one and two weeks later if no response is received. If there is no response, it is assumed that the participant has missed the data collection time and contact re-commences at the next follow-up time. If a participant cannot be contacted after three consecutive communication attempts, it is assumed that they are lost-to-follow-up. A grocery store gift card is provided to participants who complete the study. An overview of study measurements and time points can be found in Table 1.

**Table 1:**
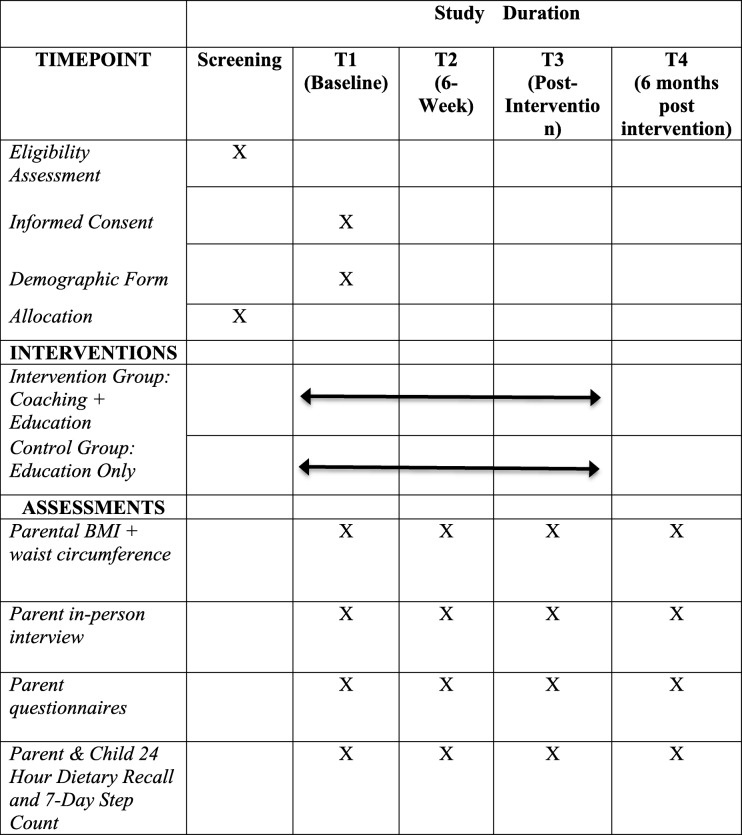
Study schedule of enrolment, interventions, and assessments

### Health education sessions for parents

Participants in the control group receive 6 online health education sessions, with three sessions focused on PA (i.e., benefits of PA; guidelines; sedentary behaviour; sleep; physical literacy; ideas for increasing PA in daily lives of parent and child; and local resources to help increase PA), and three sessions focused on nutrition (i.e., understanding nutrients and nutrition labels; eating with children; positive food environments; challenges to healthy eating; and healthy eating on a budget). The research team, who has collective expertise in each area, created and reviewed these sessions.

The online health education sessions are open to participants upon entry to the study, and parents are asked to engage in their next lesson approximately 7–10 days after their previous one. As this is intended to be a person-focused study, online lessons are being used to best accommodate the realities of each participant’s schedule. The completion of each lesson is being tracked by participant login and duration, and by a “What is the most important lesson I took from this session?” question that they are asked to complete and submit at the end of each module.

### Co-active coaching plus health education intervention

Participants in the intervention group receive the same six health education sessions described above, plus Co-Active coaching. Parent participants create a schedule with their coach to receive nine, 20-min, one-on-one, telephone-based coaching sessions (3/month for 3 months) focusing on the agenda of the parent’s choosing. Parent participants are matched with a CPCC and together, they create a schedule to engage in nine, 20-min, one-on-one telephone-based coaching sessions (3/month for 3 months). Thereafter, the participant calls the CPCC at the pre-arranged time and at the start of the conversation the client is responsible for identifying the agenda on which they want to focus during that session. The coach has been asked to use only their CPCC skills, which include but are not limited to asking genuinely curious open-ended questions, reflecting back what the participant says, acknowledging the experience that the participant shares, and championing their progress. For a full review of the Co-active coaching approach, see Co-Active Coaching: Changing Business, Transforming Lives [36].

### Measures

At baseline, the parent participant is asked to complete demographic information forms on behalf of themself and their child. Both groups complete the same assessments at each follow-up period and these consist of BMI (height and weight), waist circumference, step count, dietary intake, standardized and previously validated measures (a detailed list is included below), and open-ended questions to glean parents’ perceptions of how the intervention is impacting them and their child (any domain the parent chooses to discuss).

### Primary outcome measures

#### 7-day step count

Yamax Digi-Walker SW-650 pedometers are provided to participants (for both parent and child), as this model is used frequently in research [59, 60]. The Digi-Walker SW-650 records steps, calculates distance travelled based on individual stride length, and has a stopwatch. For the purpose of this study, only step-count data are recorded. Participants are asked to wear the pedometer for all waking hours except when swimming or bathing, and to reset it to zero each morning, over the course of one week (7 days; [59]) at all measurement time points.

#### 24-multi-pass recall

This tool is typically conducted via in-person or telephone interview; however, for the purposes of this study, dietary intake data is being conducted online. Arab and colleagues [61] tested the validity of web-based, 24-h recall within two ethnicities. When testing attenuation factors (λ; the degree to which correlations between dietary intake and true intake were underestimated or overestimated because of random error in reporting), the researchers found that the entire cohort reported high (λ = 0.28) attenuation factors with web-based recall. Additionally, the rate of underreporting of more than 30% of calories was low – 25 and 34%, for African Americans and Caucasians, respectively; [61]).

#### International physical activity questionnaire (IPAQ)

The IPAQ is a self-reported measure of PA that has been validated, and deemed acceptable for monitoring levels of physical activity, among 18–65 year olds in diverse settings [62]. Participants are asked how many days per week, and how many minutes per day they walked and engaged in moderate-intensity and vigorous-intensity activities during the past week [62]. Sedentary behaviours are assessed by asking about sedentary time accumulated from traveling, at work, watching television, and using a computer at home and at leisure; [62]). Test-retest reliability data for the long IPAQ questionnaire show Spearman correlation coefficients around 0.80, indicating very good repeatability, and criterion validity correlations ranged from 0.14 to 0.53, with a median of 0.30 [62].

#### Treatment self-regulation questionnaire (TSRQ)

The TSRQ [63] is used to determine why participants engage or would engage in healthy behaviour (i.e., their motivations). Responses are ranked on a 7-point Likert scale ranging from not at all true (1) to very true (7). Levesque and colleagues [64] used exploratory factor analysis (EFA) and confirmatory factor analysis (CFA) to test the validity of the 15-item TSRQ factorial structure. The researchers hypothesized that a 4-factor structure would emerge with: autonomous (i.e., self-determined) motivation, introjection (i.e., behaviours that have been partially taken in by the person, and are performed to avoid feeling guilty or ego involved), external (i.e., behaviour that is performed in order to obtain a reward or to avoid negative consequences), and amotivation (i.e., absence of motivation) factors [64]. The TSRQ has been validated previously with acceptable internal consistency (α > .73), and established as a useful assessment tool across various settings and for different health behaviours (e.g. tobacco, diet, and exercise; [64]).

#### In-person interviews

In-person, semi-structured interviews, exploring parents’ experiences and perceptions of the program, are conducted at each follow-up point. All interviews are voice-recorded and transcribed verbatim.

### Secondary outcome measures

#### Body mass index (BMI)

Parental height and weight is measured at baseline and each follow-up point to track changes in BMI over time.

#### Multi-dimensional scale of perceived social support (MSPSS)

The MSPSS [65] is a 12-item scale designed to measure perceived adequacy of support from family, friends, and significant others [65]. It uses a 7-point Likert scale, ranging from disagree (1) to very strongly agree (7). When assessing psychometric properties of the scale, Zimet and colleagues [66] found relatively high levels of mean support in the three sample groups they studied (6.01, 5.60, and 5.58), and an internal consistency (Cronbach’s alpha) ranging from α = 0.84 to 0.92 for the overall scale. The researchers also assessed the validity of the family and significant other subscales using multivariate analysis of variance (MANOVA) and found that both scales were significant [66].

#### Rosenberg self-esteem scale (RSE)

The purpose of the 10-item, uni-dimensional RSE [67] is to measure both positive and negative feelings about self-esteem. The RSE is comprised of a Guttman scale, using 4 response categories (strongly disagree, disagree, agree, strongly agree), and is scored on a metric ranging from 0 (poor) to 30 (excellent; [68]). Item convergent validity is generally considered satisfactory if an item correlates r ≥ .40 with its hypothesized scale after correction for overlap; the RSE met this criterion for all items overall and across subgroups [68]. Internal consistency of the scale was α = 0.91 [68].

#### Weight efficacy lifestyle (WEL) questionnaire

The WEL Questionnaire [69] consists of 20-items, which ask participants to rate their confidence about being able to successfully resist the desire to eat, using a 10-point Likert scale ranging from not confident (0) to very confident (9). The internal consistency of the scale ranged from α = 0.70 to 0.90.

#### Self-efficacy for overcoming barriers

These scales assess self-efficacy for performing PA and nutrition behaviours [70]. Self-efficacy for PA is a 12-item measure assessing how confident individuals feel (on a scale of 0–100) when overcoming barriers to being physically active. Participants also completed an 11-item measure to assess how confident individuals feel, on a scale of 0 (not confident at all) to 100 (very confident) when facing barriers to eating a well-balanced diet. Previous research supports the reliability of these scales, with alpha coefficients ranging from 0.73 to 0.95 [47, 49, 70]

#### Eating self-efficacy scale (ESES)

To further assess self-efficacy and eating behaviour, the 25-item ESES [71] is being utilized. Responses are ranked on a 7-point Likert scale ranging from no difficulty controlling eating (1) to most difficulty controlling eating (7). The ESES had a high internal consistency (α = 0.92), and the test-retest reliability over a 7-week period was also acceptable (r = 0.70, *p* < 0.001; [71]).

#### Generalized self-efficacy (GSE) scale

The GSE [72] is a 10-item scale that assesses participants’ coping ability across a wide range of demanding or novel situations [73]. It uses a Likert scale ranging from 1 (not at all true) to 4 (very true). The GSE has been used in a variety of research studies, typically yielding internal consistencies between α = 0.75 to 0.91, and a test-retest reliability (over a half-year period) of r = 0.67 [73]. When tested through an online platform, the GSE had an internal consistency of α = 0.87 (based on *n* = 1314 participants with complete data; [73]).

#### Short-form 36 (SF)-36

The self-administered 36-item SF-36 [74] is used to measure health on eight multi-item dimensions, covering functional status, well-being, and overall evaluation of health [75]. The SF-36 had an internal consistency of α > 0.85, and test-retest reliability was conducted over a two week interval and, for all dimensions, 91–98% of cases lay within the 95% confidence interval constructed for a normal distribution [75].

### Data analysis

SPSS (version 24) will be used to conduct a repeated measures MANCOVA to assess differences within groups, and a two-way ANCOVA to assess differences between groups, where baseline data will be the covariate, with differences analyzed using the post-hoc Least Square Differences method. As socioeconomic status (SES) has an impact on nearly all health issues, including obesity, and understanding its impact in the proposed project is necessary, a general linear model utilizing SES as covariate will be completed. Classification for SES will be based on education and low income cut off scores (LICO) utilizing Statistics Canada’s definition. The semi-structured interview responses from parents, to determine changes in family health behaviours and overall program experiences, will be analyzed in NVivo by two independent researchers. Inductive content analysis (a method in which patterns, themes, and categories emerge from the data, without an existing framework; [76]) will be employed to identify feedback themes. This process involves open coding, in which data is read through several times, and as many headings as necessary are written down to describe all aspects of the content [77]. The headings are then collected on a coding sheet, and categories are grouped and sorted into higher order headings [78, 79]. Data are classified into groups in order to help describe a phenomenon, increase understanding, and generate knowledge [80]. Once categories are decided upon, the two researchers compare headings and come to consensus on final themes, with the support of NVivo software.

## Discussion

This protocol paper has detailed a methodological account of a comprehensive study, aimed at exploring the impact of a parent education compared to education plus coaching intervention, with parent and child outcomes, on promoting healthy behaviours among the parent-child dyad.

The increasing rate of overweight/obesity, and the associated adverse health conditions, in increasingly younger populations creates the need for the research outlined in this paper. Given that parents are a primary influence for their children’s PA and nutrition behaviours [1], interventions that support parents in making healthy choices should be explored and promoted further.

### Strengths and limitations

In contrast with previous parent-child interventions, to the best of the researchers’ knowledge, this is the first study that will evaluate the impact of Co-Active coaching and/or education on the family unit, via parent and child outcome measures. The use of mixed methods (i.e. using both qualitative and quantitative measures) ensures that the researchers will gain an in-depth understanding of participants’ experiences with both the program and process of developing healthy behaviours. In addition, the randomized controlled trial design, use of a comparison group, and incorporation of several previously validated tools also serve as strengths in this study. Interventions that are based on the foundation of health promotion (i.e. enabling individuals to improve and increase control over their own—and in this case, their family’s—health [81]) have been shown to result in successful behaviour change [35]. This program employs education and coaching, both of which encourage participants to develop skills that will allow them to increase control over their health decisions and environments [82]. Another strength of the study is the selected coaching method in that all CPCCs involved are trained in the same manner; meaning participants in the coaching group are receiving similar strategies to help target their areas of concern [82].

A limitation of this study may be the number of participants who do not complete the program; researchers have reported participants in control conditions of lifestyle interventions are more likely to drop out [82, 83]. To counteract this, and encourage full participation, the researchers remain in contact with participants throughout the study (via email reminders to complete assessments, and in-person follow-ups). A further limitation of this study may be low participation of fathers; a review examining the involvement of fathers in pediatric obesity treatment and prevention programs with parental involvement outlined that, out of 213 included RCTs, only 6% of participants were fathers [84]. The researchers are recruiting parents using many different methods, in hopes of reaching both mothers and fathers.

## Conclusion

All of the pertinent information necessary to develop and implement a parent-child intervention has been addressed including study design, population rationale, recruitment methods, outcome measure descriptions, intervention procedures, and data collection and analysis. The expected findings from this research will provide important insights into the impact of coaching on parents with overweight/obesity, and its applications to the family unit. From conducting this study, the researchers aspire to learn effective methods to support parents and children in developing and maintaining positive nutrition and PA habits. If effective, this intervention approach can be applied more broadly through public health interventionists to parent and child populations in hopes of reducing obesity-promoting behaviours within both individuals and their families. This program is currently on going; the researchers’ intention and goal is to make the results available, via peer-reviewed publications, in 2019.

## Abbreviations

BMI: Body Mass Index
CVD: cardiovascular disease
ESES: Eating Self-Efficacy Scale
GSE: Generalized Self-Efficacy (Scale)
IPAQ: International Physical Activity Questionnaire
LICO: Low-income cut off scores
MSPSS: Multi-dimensional Scale of Perceived Social Support
MVPA: moderate-to-vigorous physical activity
PA: Physical activity
RSE: Rosenberg Self-Esteem Scale
SF-36: Short-Form 36
TSRQ: Treatment Self-Regulation Questionnaire
WEL: Weight Efficacy Lifestyle (Questionnaire)
